# Glyphosate exposure deteriorates oocyte meiotic maturation via induction of organelle dysfunctions in pigs

**DOI:** 10.1186/s40104-022-00732-0

**Published:** 2022-07-08

**Authors:** Chunhua Xing, Shun Chen, Yue Wang, Zhennan Pan, Yuanjing Zou, Shaochen Sun, Zili Ren, Yu Zhang

**Affiliations:** 1 College of Animal Science and Technology, Nanjing Agricultural University, Nanjing, 210095 China; 2 College of Animal Science, Tibet Agricultural and Animal Husbandry University, Linzhi, 860000 Tibet China

**Keywords:** Endoplasmic reticulum, Glyphosate, Lysosome, Mitochondria, Organelles, Oocyte maturation

## Abstract

**Background:**

Recently, defects in mammalian oocytes maturation induced by environmental pollution results in the decreasing animal reproduction. Animal exposed to glyphosate is largely unavoidable because glyphosate is one of the most widely used herbicide worldwide due to its high-efficiency and broad-spectrum effects, which causes glyphosate an environmental contaminant found in soil, water and food. During the last few years, the growing and wider use of glyphosate has raised great concerns about its effects of reproductive toxicity. In this study, using porcine models, we investigated effects of glyphosate on organelle functions during oocyte meiosis.

**Results:**

The results showed glyphosate exposure disrupted porcine oocyte maturation. Expression levels of cumulus expansion-related genes were interfered, further indicating the meiotic defects. The damaging effects were mediated by destruction of mitochondrial distribution and functions, which induced ROS accumulation and oxidative stress, also indicated by the decreased mRNA expression of related antioxidant enzyme genes. We also found an interference of endoplasmic reticulum (ER) distribution, disturbance of Ca^2+^ homeostasis, as well as fluctuation of ER stress, showing with the reduced ER stress-related mRNA or protein expression, which could indicate the dysfunction of ER for protein processing and signal transduction in glyphosate-exposed oocytes. Moreover, glyphosate exposure induced the disruption of lysosome function for autophagy, showing with the decrease of LAMP2 expression and autophagy-related genes mRNA expression. Additionally, our data showed the distribution of Golgi apparatus and the functions of ribosome were disturbed after glyphosate exposure, which might affect protein synthesis and transport.

**Conclusions:**

Collectively, our study showed that exposed to glyphosate could affect animal reproduction by compromising the quality of oocytes through its wide toxic effects on organelle functions.

## Background

Glyphosate is becoming one of the most widely used herbicide worldwide because of its high-efficiency and broad-spectrum effects [1]. With the pollution of soil, water, and plants, glyphosate have brought potential harm to the ecological environment and animal health [2, 3]. Recently, the previous studies suggested the genetic toxicity, hepatotoxicity, neurotoxicity of glyphosate. Moreover, glyphosate has harmful effects on the reproductive system. Glyphosate exposure damages sperm cells from healthy individuals, which show less motility and viability, and a decrease in the potential of mitochondrial membrane [4]. Glyphosate also compromises oocyte maturation by disrupting spindle organization and chromosome alignment, as well as causing oxidative stress and apoptosis in mice [5]. Additionally, glyphosate induces the reduction of both blastocyst rate and blastomere number per blastocyst in pigs, which might attribute to the impaired oocyte quality [6]. However, the toxic effects of glyphosate on porcine oocytes maturation are still unknown.

Following meiosis resumption as marked by germinal vesicle breakdown (GVBD) in response to hormones, fully grown oocytes organize the mitotic spindle, extrude half of their chromosomes into the polar body and progress up to the arrested metaphase II (MII). First polar body (PBI) extrusion is often cited as the major indicator of oocyte maturation. Additionally, the surrounding cumulus expansion is a necessary condition for oocyte meiotic maturation and development, particularly for porcine oocytes [7]. It has been reported that cumulus expansion depends on the normal expression of several genes in cumulus of cumulus-oocyte complexes (COCs), including hyaluronan synthase 2 (*HAS2*), tumor necrosis factor alpha-induced protein 6 (*TNFAIP6*), prostaglandin-endoperoxide synthase 1 (*PTGS1*), prostaglandin-endoperoxide synthase 2 (*PTGS2*) and cluster of differentiation 44 (*CD44*) [8, 9]. Therefore, the degree of cumulus expansion and the expression of cumulus expansion-related genes may be helpful to evaluate oocyte maturation.

During meiotic maturation, the substantial production and storage of the novel proteins play important roles in oocyte maturation [10]. Mitochondria functions in providing energy for proteins production, which is believed to be one of the leading determinant of oocyte quality [11]. In addition, mitochondria are major sources of intracellular ROS. The physiological level of ROS also acts as a signal transductor of successful oocyte maturation and ovulation [12]. Endoplasmic reticulum (ER) pivotal functions in biogenesis of protein folding and secretion. Also, ER is related to the spatially mitochondrial function, as linked by calcium storage and release for balance of cytoplasmic free calcium. A range of cellular disturbances may cause the accumulation of excessive misfolded proteins and loss of calcium homeostasis in ER, leading to the activation of unfold protein response and ER stress [13], which might results oocyte maturation defects. Lysosomes has long been viewed as the recycling center of cells through the endocytic and autophagic pathways [14]. The digestive functions of lysosomes could help to clear pathological cellular waste to control the quality of proteins and organelles for fundamental cellular activities [15]. Lysosome storage and degradation disorders could induce several diseases and disturbs oocyte maturation and developmental capacity [16]. The Golgi apparatus is responsible for the intracellular trafficking processes that are involved in protein synthesis and transport [17]. The disruption of Golgi functions is one of the key reasons for the failure of oocyte maturation [18]. In addition, specialized ribosomes likely regulate the translation of particular transcripts which is responsible for protein synthesis. Disorder for specialized roles of ribosomes in different ovarian follicle types and finally lead to failure of oocyte development [19].

In the present study, using porcine models, we explored the potential effects of glyphosate on organelles functions during oocyte maturation, including mitochondria, ER, lysosome, Golgi apparatus and ribosome. The results illustrated that the potential toxic mechanisms of glyphosate were mediated by a wide disruption of organelles functions, which further explained the reduced oocyte quality after glyphosate exposure.

## Methods

### Antibodies and chemicals

Mito-Tracker Red CMXRos (M7512) was purchased from Invitrogen (Carlsbad, CA, USA). Lysosome-Tracker Red (C1046), ER-Tracker Red (C1041), Golgi-Tracker Red (C1043), [dichlorofluorescein (DCFH)] diacetate (DCFHDA) Kit (S0033) and Fluo-4 AM (S1060) was purchased from Beyotime Institute of Biotechnology (Shanghai, China). Rabbit polyclonal anti-LAMP2 antibody (27823–1-AP), rabbit polyclonal anti-GRP78 antibody (11587-AP-1) and rabbit polyclonal anti-RPS3 antibody (11990–1-AP) were purchased from Proteintech (Chicago, IL, USA). Rabbit anti-GM130 antibody (ab52649) was purchased from Abcam (Cambridge, UK). Alexa FITC-goat anti-rabbit IgG (ZF0311) was from Zhongshan Golden Bridge-Biotechnology Co., Ltd. (Beijing, China). All other chemicals used in this study were purchased from Sigma (St. Louis, MO, USA), unless otherwise indicated.

### COCs collection and in vitro maturation

All animal experiments were carried out according to Nanjing Agricultural University Care and Use Committee Guidelines. Porcine ovaries were collected from a local slaughterhouse (Nanjing, China), placed in sterile physiological saline including 800 IU/m gentamicin in a thermos bottle and transported to the laboratory within 2 h. After being washed with sterile phosphate-buffer saline twice, the COCs were sucked from the 3–6 mm follicles with 20-gauge needles and oocytes with intact and compact cumulus mass were selected for maturation.

The in vitro maturation (IVM) medium was composed of TCM-199 (M7653, St. Louis, MO, USA) supplement with 3.05 mmol/L D-glucose (G7021, St. Louis, MO, USA), 75 μg/mL penicillin (Lot No: S090615, HuaLu Group (Jining, China)), 50 μg/mL streptomycin (Lot No: 10072901, ReYoung Co., Ltd. (Zibo, China)), 0.91 mmol/L sodium pyruvate (11360–070, Gibico Life Technologies (Grand Island, NY, USA)), 0.57 mmol/L cysteine (C7880, St. Louis, MO, USA), 10% (v/v) porcine follicular fluid (PFF), 10 ng/mL epidermal growth factor (EGF) (E4127, St. Louis, MO, USA), 10 IU/mL pregnant mare serum gonadotropin (PMSG) (A006, Ningbo second hormone factory (Cixi, China)) and human chorionic gonadotropin (hCG) (A001–2, Ningbo second hormone factory (Cixi, China)). The total of 60–70 selected germinal vesicle (GV) COCs in each group were transferred into a four-well dish (Nunc, Roskilde, Denmark) including 500 μL IVM medium that is covered with 200 μL mineral oil, and cultured at 38.5 °C with 5% CO_2_ for 26–28 h to metaphase I stage (MI) or for 44–48 h to MII stage. The surrounding cumulus cells were gently stripped from COCs with a fine-bore pipette in 0.1% hyaluronidase (in TCM-199), and denuded oocytes were obtained for subsequent analysis.

### Analysis of oocyte nuclear maturation and cumulus cell expression

For proper judgment of oocyte nuclear maturation, COCs were subjected to IVM for 44 h. After mechanically stripping, the denuded oocytes were transferred to 100 μL TCM199 droplet, and the polar body of each oocyte was then observed under Olympus IX53 inverted microscope (Olympus Corp., Tokyo, Japan) (× 100) by rotating oocyte one by one with a thin sealed glass needle. Oocyte with an extruded polar body was judged as matured.

To assess cumulus expansion, groups of COCs were cultured for 44 h, and then, under a stereomicroscope, the degree of cumulus expansion was observed and classified. The subjective scoring system for assessment of cumulus expansion was established based on the published literatures [20, 21]. Specifically, cumulus expansion was classified into three grades. Grade A-complete expansion, the COCs are surrounded by complete loose cumulus, and the maximum diffusion diameter of these loosening cumulus was more than 3 times (including 3) of denuded oocyte diameter. Grade B-partial expansion, the outer layer cumulus cells of COCs are loose, and the maximum expansion diameter of cumulus cells is above 2 times (including 2) but less than 3 times of the oocytes. Grade C-poor expansion, the COCs are surrounded with dense cumulus cells, and the expansion diameter of cumulus cells is less than 2 times of oocytes.

### Glyphosate treatment

Glyphosate was dissolved in TCM199 medium to 10 mmol/L stock solution and then diluted in IVM medium to final concentration of 100 μmol/L, 200 μmol/L and 400 μmol/L, respectively. The sick storage of each experiment is used on the spot.

### Detection of mitochondria, ER and lysosome

The distribution of mitochondria, ER and lysosome was detected by MitoTracker Red CMXRos, ER-Tracker Red and Lyso-Tracker Red respectively. Based on the published literatures [22, 23], and meanwhile, considering the pivotal roles of organelles in metaphase I (MI) stage on successful maturation of oocyte, MI oocytes were applied in this work. For these, MI oocytes from each group were collected and stained with 500 nmol/L Mito-Tracker Red (in IVM medium), 1 μmol/L ER-Tracker or 0.5 μmol/L Lyso-Tracker for 30 min at 38.5 °C in 5% CO_2_ incubator, individually. After being washed three times in TCM199, oocytes were mounted on the glass-bottom culture dish supplemented with PBS droplet and examined under a confocal laser-scanning microscope (Zeiss LSM 900 META, Germany).

### Cytoplasmic Ca^2+^ and Golgi apparatus staining

For Ca^2+^ and Golgi staining, zona pellucida of MI oocyte was firstly removed by incubating with 1% preheated protease for 3 min. Then, oocytes were transferred to 1 mmol/L Fluo-4 AM (in IVM medium) or Golgi-Tracker (1:200 in IVM medium) for 30 min at 38.5 °C in 5% CO_2_. After being washed three times with IVM medium, oocytes were transferred to the glass-bottom culture dish supplemented with PBS droplet. The fluorescence signal was observed with a confocal laser-scanning microscope (Zeiss LSM 900 META, Germany).

### Reactive oxygen species (ROS) detection

ROS was measured with DCFH diacetate (DCFHDA) Kit. For this, living oocytes were cultured in 10 μmol/L DCFH-DA (in DPBS) for 30 min at 38.5 °C. After being washed three times in preheated culture medium, oocytes were transferred to the glass-bottom culture dish supplemented with PBS droplet and the signals from each group were detected with the same scanning settings immediately. The fluorescence intensity of each oocyte was analyzed by Zen lite 2012 and Image J software (National Institutes of Health, Bethesda, MD, USA).

### Immunofluorescence staining and confocal microscopy

Denuded oocytes from each group were immobilized in 4% paraformaldehyde for 30 min at room temperature, and then were permeabilized with 1% Triton X-100 (in PBS) at room temperature for 8–12 h. After being blocked with 1% BSA-suppled phosphate-buffer saline (PBS) for 1 h to inhibit nonspecific binding of IgG, oocytes were stained with RPS3 antibodies (1:100) overnight at 4 °C. After being washed three times in PBS containing 0.1% Tween 20 and 0.01% Triton X-100, oocytes were incubated with secondary goat anti-rabbit IgG for 1 h at room temperature. Finally, oocytes were mounted on the glass slides and then observed with Zeiss LSM 900 META.

### Western blot analysis

120 denuded MI oocytes were collected, and then lysed in 12 μL sample buffer (SDS sample buffer including 2-meracptoethanol) at 100 °C for 10 min. Proteins were separated by 10% SDS-polyacrylamide gel electrophoresis and then transferred on a poly-vinylidene fluoride membrane (Millipore, US 5 Billerica, MA, USA). After being blocked in TBST including 5% nonfat milk for 1 h at room temperature, membranes were incubated with the primary antibodies (anti-LAMP2, 1:1000; anti-GM130, 1:3000, anti-GRP78, 1:3000, anti-α-tubulin, 1:2000,) at 4 °C overnight. After being washed three times in TBST for 10 min each time, membranes were incubated with the HRP (horse radish peroxidase) conjugated goat anti-rabbit IgG (Santa Cruz, TX, USA) for 1 h. After being washed, membranes were transferred to chemiluminescence reagent (Millipore, Billerica, MA, USA). Finally, signals were visualized with an ECL Plus enhanced chemiluminescence detection system (Tanon3900, Shanghai, China). Equal protein loading was confirmed by the α-tubulin levels.

### RNA extraction and quantitative real time

Total RNA was extracted from 30 MI oocytes with a Dynabeads mRNA DIRECTTM kit (Invitrogen), and then was reversed to cDNA with a Prime-Script RT Master Mix (Takara, Japan), which was followed by storage at − 20 °C until use. Quantitative real-time PCR was performed with SYBR Green PCR Master Mix by a Quant-Studio 5 Flex Real-Time PCR System (TermoFisher, Waltham, MA, USA). Each reaction system is 20 μL, including 10 μL of Fast Universal SYBR Green Master (Rox), 0.8 μL of specific primers, 7.2 μL of water, and 2 μL cDNA sample. *GAPDH* was recognized as control, and data was calculated with 2^-^^ΔΔ^^Ct^ method. The primers are listed in Table 1.

**Table 1:** Primer sequences for RT-qPCR

Gene	Forward primer (5' to 3')	Reverse primer (5' to 3')
*CD44*	GAGGATGATATGAGCAGTGG	GGTGCGTAGTAGTCGGAAG
*HAS*	TGGCTGTACAATGCGATGTG	TGGGTGGTGTGATTTTCACC
*TNFAIP6*	TCTTCCTGTGGGAAGAGGCT	GTCCGTCTGAACAGAAGCGA
*PTGS1*	AACACGGCACACGACTACA	CTGCTTCTTCCCTTTGGTCC
*PTGS2*	ACAGGGCCATGGGGTGGACT	CCACGGCAAAGCGGAGGTGT
*RPL19*	TGCTCGAATGCCTGAGAAG	GGTACAGACTGTGATACATG
*PGC1α*	GACACAACACGGACAGAA	CACAGGTATAACGGTAGGTAA
*ATP5B*	TTGTTGGCAGTGAGCATT	AACCTGGAATGGCTGAGA
*SOD1*	ATCAAGAGAGGCACGTTGGA	TCTGCCCAAGTCATCTGGTT
*SOD2*	TCAAGGAGAAGTTGACCGCT	AGGTAATACGCATGCTCCCA
*CAT*	AGATGGACACAGGCACATGA	CCGGATGCCATAGTCAGGAT
*GPX*	CACCCAGATGAATGAGCTGC	CATGAAGTTGGGCTCGAACC
*GRP78*	GGTGGGCAAACAAAGACATT	CGCTGGTCAAAGTCTTCTCC
*ATF4*	TGAGCCCTGACTCCTATCTG	TCCAGCTCTTTACATTCGCC
*CHOP*	AGGCCTGGTATGAGGACCTG	GCTGTGCCACTTTCCTTTCA
*LAMP2*	GCTTTTGCAGCGTTGTGG	GACGAGGCAGAGCATAAGGAG
*LC3*	CCGAACCTTCGAACAGAGAG	AGGCTTGGTTAGCATTGAGC
*ATG7*	AGATTGCCTGGTGGGTGGT	GGGTGATGCTGGAGGAGTTG
*GAPDH*	AAGTTCCACGGCACAGTCAAG	CACCAGCATCACCCCATTT
*mtDNA*	ACACACCCTATAACGCCTTGCC	GGGTAGGTGCCTGCTTTCGTAG
*18S*	CCCACGGAATCGAGAAAGAG	TTGACGGAAGGGCACCA

### Assessment of cumulus expression genes

Cumulus cell expansion was assessed by the expression levels of cumulus-expansion-related genes. Having been stripped after 44 h culture, cumulus cells from 45 COCs were collected. Total RNA was extracted from these cumulus cells and then performed the quantitative real-time PCR. The expression of *RPL19* was recognized as a control gene, and data was calculated with 2^-^^ΔΔ^^Ct^ method. The primers are listed in Table 1.

### Mitochondrial DNA (mtDNA) copy number analysis

Total DNA from 50 MI oocytes was first extracted with DNA extracted Kit (Sangon, Shanghai, China), and then the expression of mtDNA number was detected by quantitative real time-PCR. The expression levels of genes were calculated following the 2^-^^ΔΔ^^Ct^ method, and *18S* was conducted as a control gene. Primers were shown in Table 1.

### Fluorescence intensity analysis

For measurement of fluorescence intensity, oocytes from control and treatment groups were placed in near areas on the same glass slide, and then, were conducted in parallel and under identical conditions with the same confocal microscope settings without alteration. Image J software (U.S. National Institutes of Health, Bethesda, MD, USA) was used to measure the fluorescence intensity. For this, the average fluorescence intensity per unit area within the region of interest was calculated. Then, the mean values of all measurements among groups were conducted to compare the final average intensity. The fluorescence intensity of control groups was recorded as 1.

### Statistical analysis

Each experiment was performed at least three independent biological replicates with more than 30 oocytes in each group. Data were expressed as mean ± SEMs and analyzed by *t*-test or one-way ANOVA between controls and treatments with GraphPad Prism 5 software (GraphPad, San Diego, CA). Value of *P* < 0.05 was considered as statistically significant.

## Results

### Glyphosate exposure affects porcine oocyte maturation

**Fig. 1:**
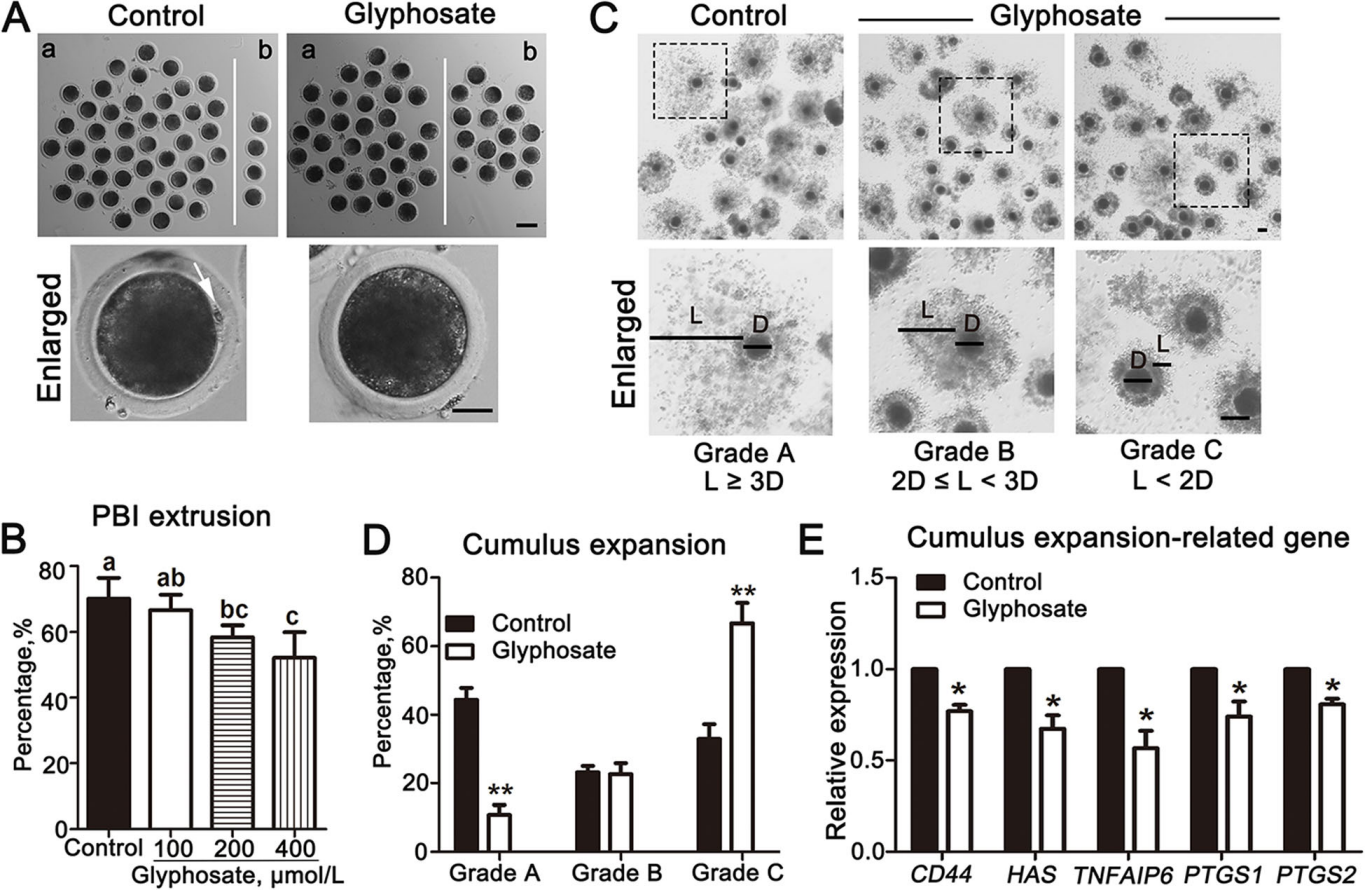
Glyphosate exposure affects porcine oocyte maturation. **A** Representative morphology of oocytes maturation after 400 μmol/L glyphosate exposure. a: porcine oocytes with polar body. b: porcine oocytes without polar body. Arrow indicated PBI. Bar = 100 μm, Enlarge: Bar = 30 μm. **B** Rate of polar body extrusion in control and different concentrations of the glyphosate-exposed groups (100, 200 and 400 μmol/L). Different letters, significant difference (*P* < 0.05). **C** Representative images of COCs with 3 deferent expansion degrees. Bar = 100 μm. Glyphosate: 400 μmol/L. **D** Relative expansion of cumulus was analyzed in the control and 400 μmol/L glyphosate groups. Grade A: complete expansion; Grade B: partial expansion; Grade C: poor expansion. **, *P* < 0.01. Glyphosate: 400 μmol/L. **E** Expression of cumulus expansion-related genes was detected in control and 400 μmol/L glyphosate groups. *, *P* < 0.05

To investigate the possible toxic effects of glyphosate on porcine oocytes, we firstly assessed oocytes maturation following different concentrations (100, 200, and 400 μmol/L) of glyphosate treatment after 46 h culture, during which most oocytes should have reached MII. PBI extrusion was assessed for judgement of oocyte maturation. As shown in Fig. 1A, PBI extrusion succeeded in most control oocytes, but failed in a considerable proportion of oocytes after glyphosate exposure. The statistical data showed that compared to the controls (70.07 ± 6.38%, *n* = 220 oocytes), the rate of polar body extrusion remarkably decreased in 200 μmol/L and 400 μmol/L glyphosate exposed oocytes (200 μmol/L: 58.45 ± 3.56%, *P* < 0.05, *n* = 258 oocytes; 400 μmol/L: 52.20 ± 7.79%, *P* < 0.01, *n* = 250 oocytes). There was no difference after 100 μmol/L glyphosate treatment (66.64 ± 4.63%, *P* > 0.05, *n* = 262 oocytes) (Fig. 1B). Thus, 400 μmol/L of glyphosate was selected for the subsequent studies.

Cumulus cells expansion was then examined to further assess effects of glyphosate on the developmental capacity of the oocytes. As shown in Fig. 1C, most of the cumulus were good expanded in the control, whereas numerous poor expanded cumulus were observed after 400 μmol/L glyphosate exposure. For visually analyzing COCs expansion, we also quantified the expansion degrees of cumulus. The diameter of oocytes (recorded as D) as well as the cumulus diffusion diameter (recorded as L) was quantified along the long axis of oocytes. The expansion degrees of cumulus were classified as Grade A (complete expansion), Grade B (partial expansion) and Grade C (poor expansion) by evaluating the relative expansion of cumulus (L/D) in both glyphosate-exposed and control groups. As shown in Fig. 1D, the percentage of COCs in Grade A was significantly decreased after 400 μmol/L glyphosate exposure (control: 44.36 ± 3.46%, *n* = 105 COCs vs. glyphosate: 10.78 ± 2.91%, *n* = 128 COCs, *P* < 0.01), and meanwhile, the percentage of COCs in Grade C was significantly increased after glyphosate exposure compared with the control (control: 32.87 ± 4.34%, *n* = 105 COCs vs. glyphosate: 66.62 ± 5.92%, *n* = 128 COCs, *P* < 0.01); however, there was no difference in Grade B between glyphosate-exposed and the control groups (control: 23.18 ± 1.87%, *n* = 105 COCs vs. glyphosate: 22.60 ± 3.23%, *n* = 128 COCs, *P* > 0.05). To verify this, then, the expression levels of cumulus expansion-related genes were observed. As shown in Fig. 1E, the expression levels of cumulus expansion-related genes significantly decreased after 400 μmol/L glyphosate exposure compared with the control (*CD44*, control: 1.00 vs. glyphosate: 0.77 ± 0.03, *P* < 0.05, *n* = 135 COCs; *HAS*, control: 1.00 vs. glyphosate: 0.67 ± 0.07, *P* < 0.05, *n* = 135 COCs; *TNFAIP6*, control: 1.00 vs. glyphosate: 0.57 ± 0.09, *P* < 0.05, *n* = 135 COCs; *PTGS1*, control: 1.00 vs. glyphosate: 0.74 ± 0.08, *P* < 0.05, *n* = 135 COCs; *PTGS2*, control: 1.00 vs. glyphosate: 0.81 ± 0.03, *P* < 0.05, *n* = 135 COCs). Overall, these results suggested that glyphosate exposure might disrupt oocyte maturation in porcine.

### Glyphosate exposure impairs mitochondria functions in porcine oocytes

**Fig. 2:**
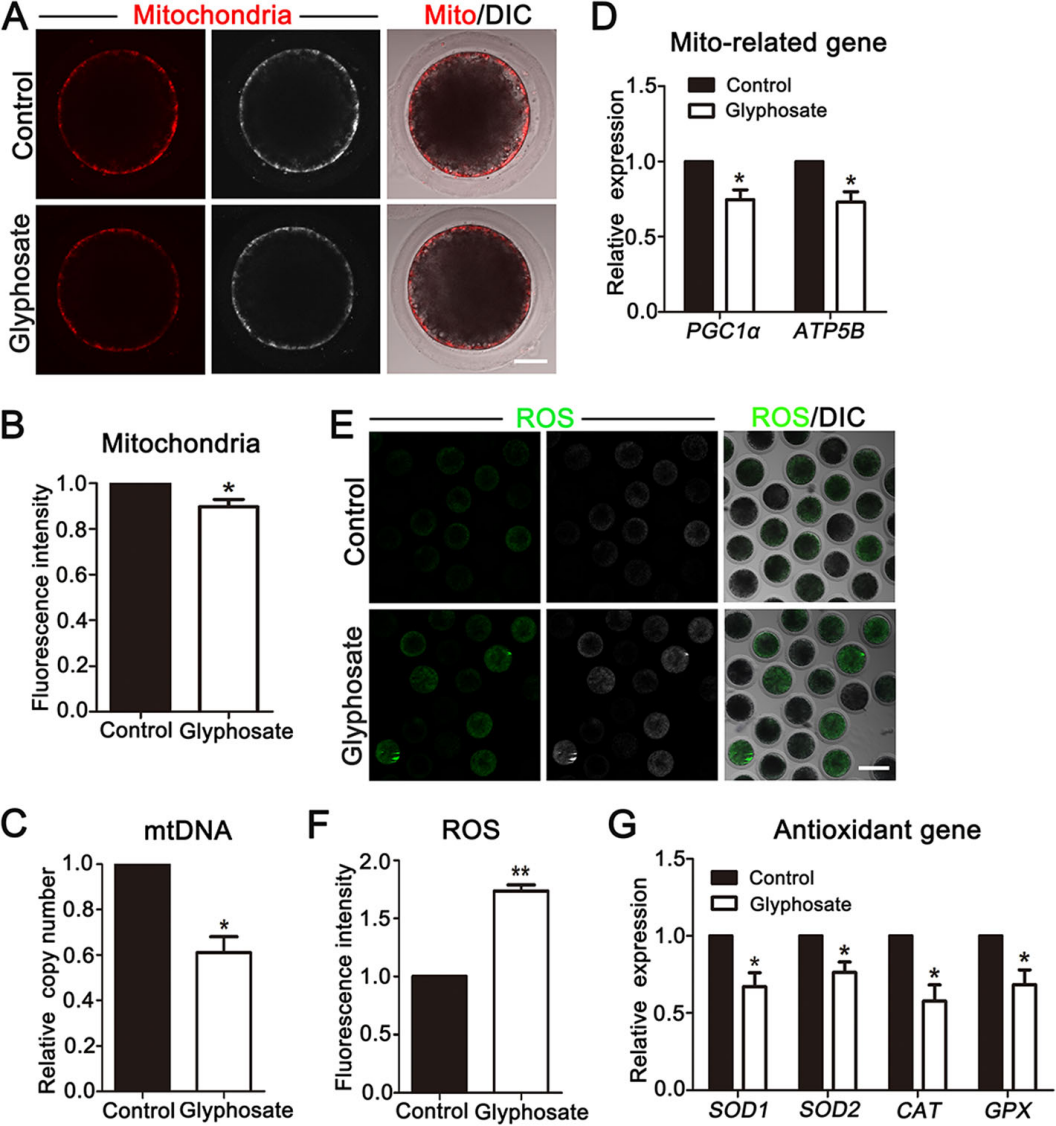
Glyphosate exposure impairs mitochondria functions in porcine oocytes. **A** Representative images of mitochondria in control and 400 μmol/L glyphosate-exposed oocytes. Bar = 30 μm. **B** The relative fluorescence intensity of mitochondria after 400 μmol/L glyphosate exposure. *, *P* < 0.05. **C** Relative mtDNA copy number was assessed in control and 400 μmol/L glyphosate-exposed oocytes. *, *P* < 0.05. **D** Expression of mitochondrial-related genes was detected by RT-PCR in the control and 400 μmol/L glyphosate-exposed oocytes. *, *P* < 0.05. **E** Representative images of ROS signals in control and 400 μmol/L glyphosate-exposed oocytes. Bar = 100 μm. **F** The relative fluorescence intensity of ROS was analyzed in control and 400 μmol/L glyphosate-exposed oocytes. **, *P* < 0.01. **G** Relative expression of antioxidant-related genes in control and 400 μmol/L glyphosate-exposed oocytes. *, *P* < 0.05

Proper mitochondria functions are critical for oocyte maturation. To explore how glyphosate exposure affects oocyte maturation, we then assessed the mitochondrial dynamics. Firstly, mitochondrial distribution was examined by Mito-Tracker staining. As shown in Fig. 2A, mitochondria were mainly located in the cortical region of control oocytes. However, the fluorescence signals of mitochondria at the cortex decreased in glyphosate-exposed group. Analysis of the fluorescence intensity also confirmed this (control:1.00, *n* = 60 oocytes vs. glyphosate: 0.89 ± 0.03, *P* < 0.05, *n* = 54 oocytes) (Fig. 2B). We then detected mitochondrial DNA copy number to evaluate mitochondrial functions. Our results showed that the relative mitochondrial DNA copy number significantly decreased in glyphosate-exposed oocytes (0.61 ± 0.07, *P* < 0.05, *n* = 150 oocytes) compared with the controls (1.00, *n* = 150 oocytes) (Fig. 2C). Next, with quantitative real time-PCR, we detected the expression levels of mitochondria-related genes, *PGC1α* (Peroxisome proliferator activated receptor coactivator 1 alpha) and *ATP5B* (ATP synthase, H^+^ transporting, mitochondrial F1 complex, beta polypeptide). As shown in Fig. 2D, the expression levels of *PGC1α* and *ATP5B* decreased in glyphosate treatment oocytes (*PGC1α*, control: 1.00 vs. glyphosate: 0.75 ± 0.07, *P* < 0.05, *n* = 90 oocytes; *ATP5B*, control: 1.00 vs. glyphosate: 0.73 ± 0.06, *P* < 0.05, *n* = 90 oocytes). Given that mitochondrial dysfunction might induce excessive ROS accumulation for inducing oxidative stress, we also detected the ROS level by DCFH staining. As shown in Fig. 2E, ROS signals in glyphosate-exposed oocytes were statically different from the controls. In addition, the quantitative analysis of ROS fluorescence intensity also confirmed this. (control: 1.00, *n* = 80 oocytes vs. glyphosate: 1.74 ± 0.05, *n* = 76 oocytes, *P* < 0.01) (Fig. 2F). Furthermore, the expression of antioxidant genes, including superoxide dismutase 1 (*SOD1*)*,* superoxide dismutase 2 (*SOD2*), catalase (*CAT*) and glutathione peroxidase (*GPX*), was also determined by quantitative real time-PCR. Our data showed that the levels of these genes were all downregulated after glyphosate exposure (*SOD1*, control: 1.00 vs. glyphosate: 0.67 ± 0.09, *P* < 0.05, *n* = 90 oocytes; *SOD2*, control: 1.00 vs. glyphosate: 0.76 ± 0.07, *P* < 0.05, *n* = 90 oocytes; *CAT*, control: 1.00 vs. glyphosate: 0.58 ± 0.10, *P* < 0.05, *n* = 90 oocytes; *GPX*, control: 1.00 vs. glyphosate: 0.68 ± 0.09, *P* < 0.05, *n* = 90 oocytes) (Fig. 2G). Overall, these results suggested that glyphosate induced mitochondrial dysfunction and oxidative stress in porcine oocytes.

### Glyphosate exposure affects ER functions in porcine oocytes

**Fig. 3:**
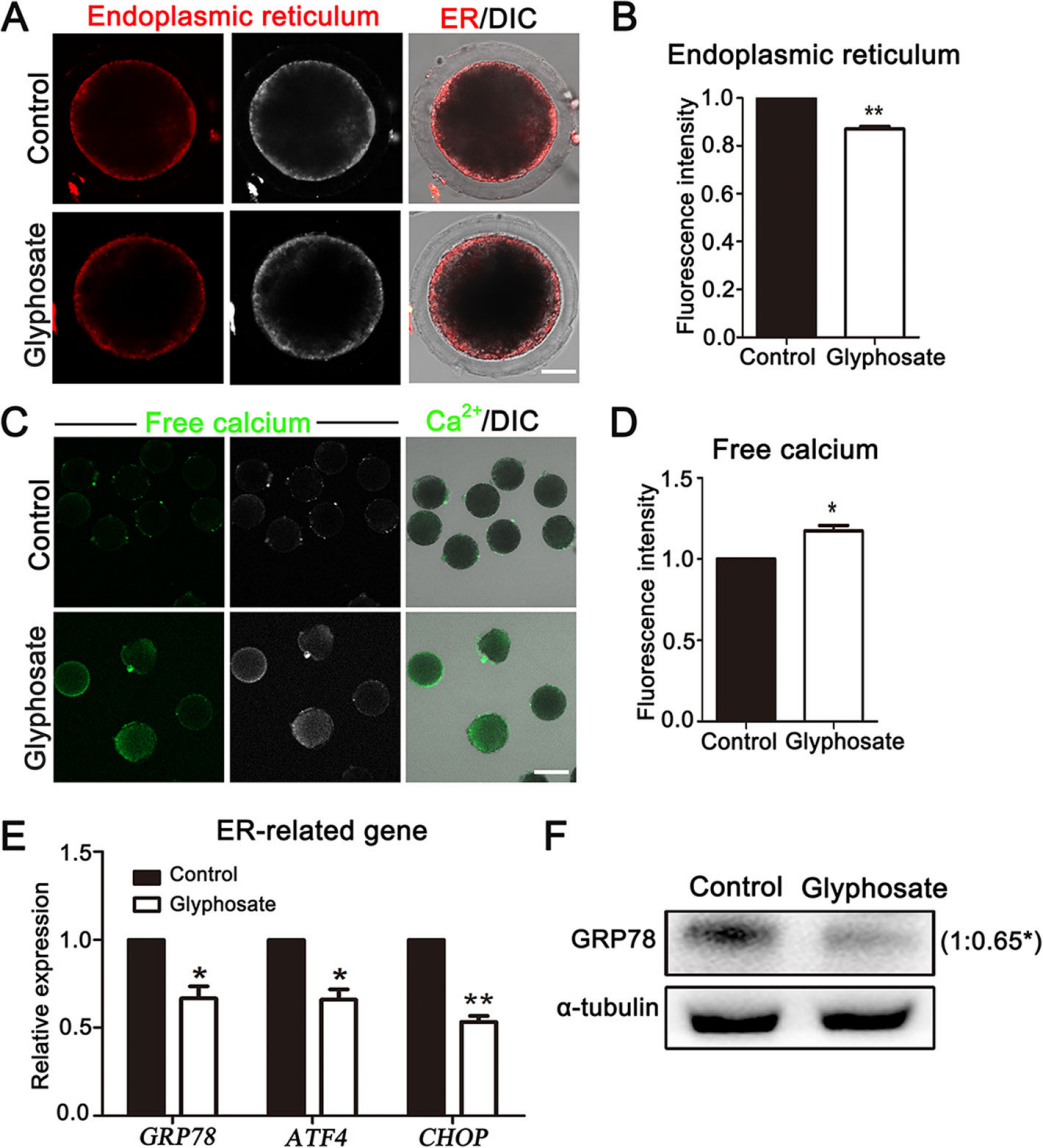
Glyphosate exposure affects ER functions in porcine oocytes. **A** Representative images of ER distribution in control and 400 μmol/L glyphosate exposure oocytes. Bar = 30 μm. **B** The relative florescence intensity of ER in control and 400 μmol/L glyphosate-exposed oocytes. **, *P* < 0.01. **C** Representative images of Ca^2+^ in control and 400 μmol/L glyphosate-exposed groups. Bar = 30 μm. **D** The relative florescence intensity of Ca^2+^ after 400 μmol/L glyphosate treatment. *, *P* < 0.05. **E** Relative expression of ER-related genes was measured in control and 400 μmol/L glyphosate-exposed oocytes. *, *P* < 0.05; **, *P* < 0.01. **F** The protein expression of GRP78 in the control and 400 μmol/L glyphosate-exposed oocytes. *, *P* < 0.05

The pivotal functions of ER are involved in biogenesis of protein folding and secretion as well as homeostasis of cytoplasmic Ca^2+^ during oocyte maturation, which prompted us to investigate functions of ER after glyphosate exposure. We first detected ER distribution by ER-Tracker staining. As shown in Fig. 3A, the fluorescence signals of ER were distributed in the cortical region of control oocytes, but decreased after glyphosate exposure, which was also confirmed by the fluorescence intensity analysis (control: 1.00, *n* = 41 oocytes vs. glyphosate: 0.87 ± 0.01%, *n* = 35 oocytes, *P* < 0.01) (Fig. 3B). In addition, the results showed signals of Ca^2+^ in glyphosate-exposed oocytes were much stronger than those of controls (Fig. 3C), intensity analysis also confirmed (control: 1.00, *n* = 70 oocyte vs. glyphosate: 1.17 ± 0.03%, *n* = 65 oocytes, *P* < 0.05) (Fig. 3D). Moreover, we also detected mRNA expression levels of glucose-regulated protein 78 (*GRP78*), activating transcription factor 4 (*ATF4*) and C/EBP homologous protein (*CHOP*) that were involved in ER functions. Our data showed that the expression levels of those genes reduced in the glyphosate exposure groups compared with those of controls (*GRP78*, control: 1.00 vs. glyphosate: 0.67 ± 0.07, *P* < 0.05, *n* = 90 oocytes; *ATF4*, control: 1.00, vs. glyphosate: 0.66 ± 0.05, *P* < 0.05, *n* = 90 oocytes; *CHOP*, control: 1.00 vs. glyphosate: 0.53 ± 0.03, *P* < 0.01, *n* = 90 oocytes) (Fig. 3E). To confirm whether ER stress was induced in glyphosate-exposed oocytes, the protein expression of GRP78 was also assessed. We found that the expression of GRP78 significantly decreased after glyphosate exposure compared with the control groups, and the quantitative analysis also confirmed this (control: 1.00 vs. glyphosate: 0.65 ± 0.06%, *P* < 0.05, *n* = 360 oocytes) (Fig. 3F). Taken together, these results suggested that glyphosate exposure might induce ER dysfunctions in porcine oocytes.

### Glyphosate exposure disrupts lysosome functions in porcine oocytes

**Fig. 4:**
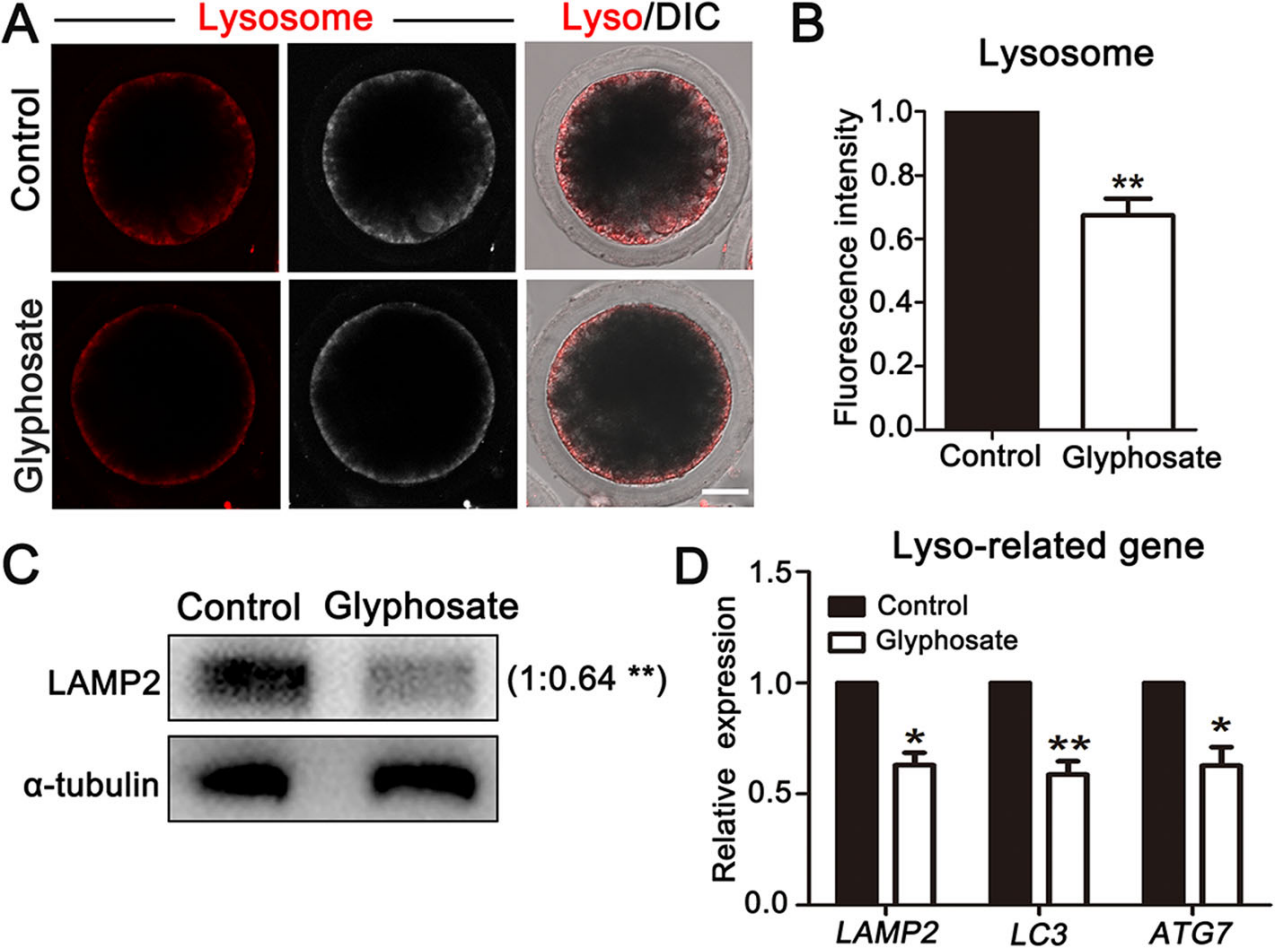
Glyphosate exposure disrupts lysosome functions in porcine oocytes. **A** Representative images of lysosome distribution in control and 400 μmol/L glyphosate treatment groups. Bar = 30 μm. **B** The relative florescence intensity of lysosome after 400 μmol/L glyphosate treatment. **, *P* < 0.01. **C** Western blot analysis for the protein LAMP2 expression in control and 400 μmol/L glyphosate exposure groups. **, *P* < 0.01. **D** Relative expression of lysosome-related genes was analyzed in control and 400 μmol/L glyphosate-exposed oocytes. *, *P* < 0.05; **, *P* < 0.01

As functions of lysosome in degradation of protein, nucleic acid, polysaccharide and organelle are responsible for recycling degradation products to promote metabolism during oocyte maturation, we also investigated whether glyphosate affects lysosome functions. Firstly, we measured the distribution of lysosomes by Lyso-Tracker, and found that lysosomes were located at cortex of porcine oocytes, whereas the fluorescence signals markedly dropped in glyphosate exposed-oocytes (Fig. 4A), which was also revealed by the fluorescence intensity data (control: 1.00, *n* = 53 oocytes vs. glyphosate: 0.67 ± 0.05%, *n* = 50 oocytes, *P* < 0.01) (Fig. 4B). To confirmed this, we also examined the expression of lysosome marker protein, lysosome-associated membrane protein 2 (LAMP2), and the results showed a significantly decreased LAMP2 expression after glyphosate exposure compared with that of controls (control: 1.00 vs. glyphosate: 0.64 ± 0.05%, *P* < 0.01, *n* = 500 oocytes) (Fig. 4C). Furthermore, we also examined mRNA expression levels of autophagy-related genes that were involved in the important degradation functions of lysosomes. As shown in Fig. 4D, the relative mRNA expression levels of *LAMP2*, microtubule-associated protein 1 light chain 3 (*LC3*) and autophagy-related gene 7 (*ATG7*), significantly decreased after glyphosate exposure (*LAMP2*, control: 1.00 vs. glyphosate: 0.63 ± 0.05, *P* < 0.05, *n* = 90 oocytes; *LC3*, control: 1.00 vs. glyphosate: 0.59 ± 0.06, *P* < 0.01, *n* = 120 oocytes; *ATG7*, control: 1.00 vs. glyphosate: 0.63 ± 0.08, *P* < 0.05, *n* = 90 oocytes). Therefore, these data indicated that glyphosate exposure might affect lysosome functions in porcine oocytes.

### Glyphosate exposure damages distribution of Golgi apparatus and functions of ribosome in porcine oocytes

**Fig. 5:**
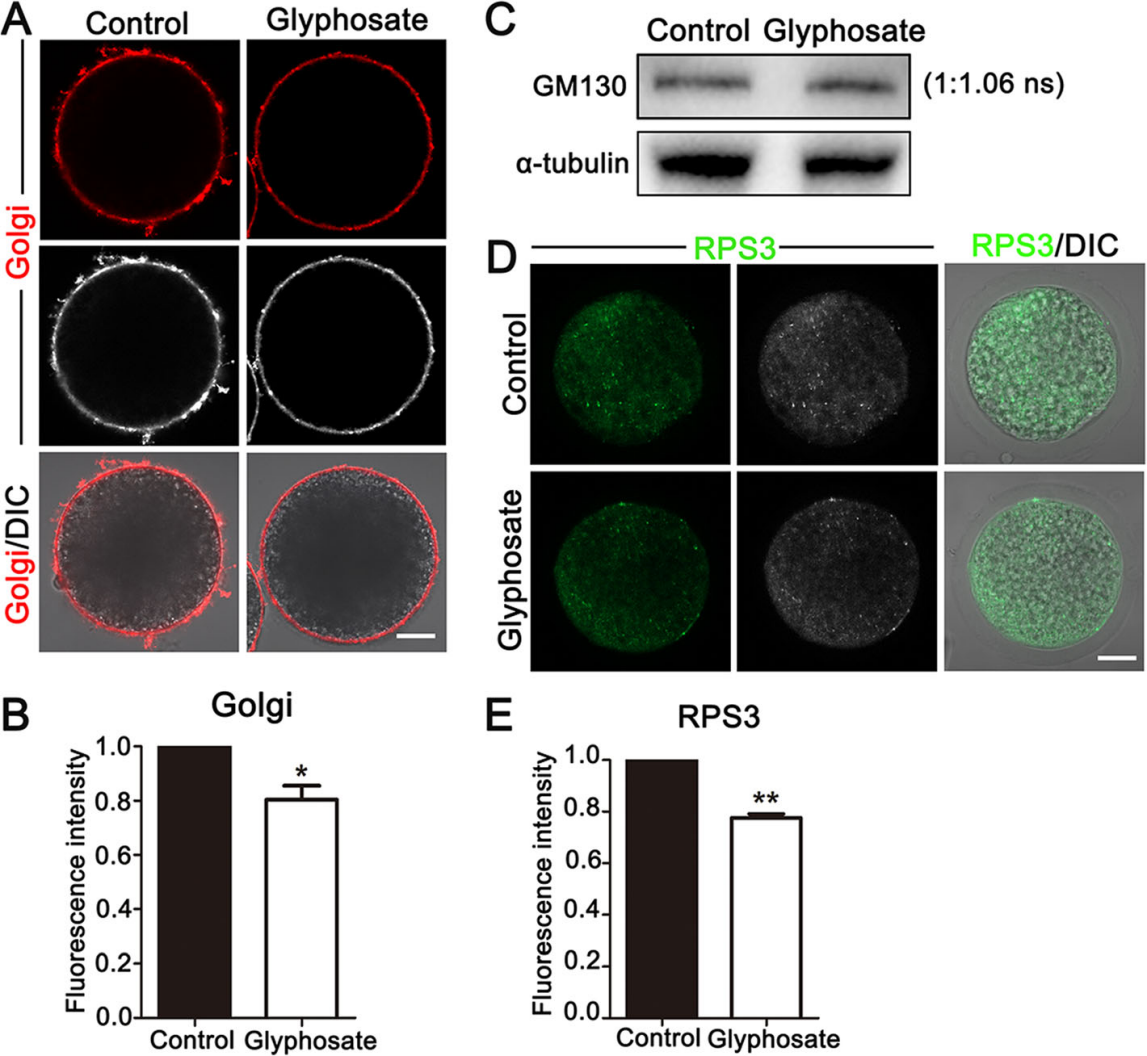
Glyphosate exposure damages Golgi apparatus distribution and ribosome functions in porcine oocytes. **A** Representative images of Golgi apparatus distribution in control and 400 μmol/L glyphosate treatment groups. Bar = 30 μm. **B** The relative florescence intensity of Golgi apparatus after 400 μmol/L glyphosate treatment. *, *P* < 0.05. **C** Western blot analysis for the protein expression of GM130 in control and 400 μmol/L glyphosate exposure groups. ns: no significance. **D** Representative images of ribosome distribution in control and 400 μmol/L glyphosate treatment groups. Bar = 30 μm. **E** The relative florescence intensity of RPS3 after 400 μmol/L glyphosate treatment. **, *P* < 0.01

During oocyte maturation, Golgi apparatus plays a pivotal role in processing, sorting and packaging proteins synthesized by ER, after that transporting them to specific parts to participate in cellular processes. To further investigate effects of glyphosate on oocytes, we also examined functions of Golgi apparatus. As shown in Fig. 5A, through Golgi-Tracker staining, Golgi showed a significant distribution at the cortex of porcine oocyte, but the fluorescence signals decreased after glyphosate exposure (control: 1.00, *n* = 62 oocytes vs. glyphosate: 0.81 ± 0.05%, *n* = 40 oocytes, *P* < 0.05) (Fig. 5B). Then, western blot was used to examine the protein expression of the Golgi marker, the Golgi matrix protein matrix protein 130 (GM130). As shown in Fig. 5C, there is no difference in the GM130 expression in control and glyphosate exposure groups. The statistical analysis of intensity also confirmed this (control: 1.00 vs. glyphosate: 1.06 ± 0.11%, *P* > 0.05, *n* = 360 oocytes). Thus, glyphosate exposure led to defects of Golgi distribution but no dysfunction. Given that ribosome is a pivotal organelle for protein synthesis during meiosis, we also detected ribosome functions. Via ribosomal protein S3 (RPS3) staining, as shown in Fig. 5D, we found the signals of RPS3 were distributed in cytoplasm evenly in control oocytes, while a significant decrease was found in glyphosate-exposed oocytes. Compared with controls (1.00, *n* = 44 oocytes), the fluorescence intensity of RPS3 significantly decreased to 0.78 ± 0.01% (*n* = 50 oocytes, *P* < 0.01) (Fig. 5E). Overall, these results indicated that glyphosate exposure might cause abnormal distribution of Golgi apparatus and dysfunction of ribosome.

## Discussion

In recent years, the pesticide glyphosate has drawn widespread attention due to their adverse effects on animal health, especially reproduction. Given that pigs are more sensitive to toxicity, pigs are one of the most ideal models to evaluate the toxicity of environmental pollutant to animals. In this study, we employed porcine oocytes to detect the toxic effects of glyphosate on organelles functions during oocytes maturation. Our results indicated that glyphosate exposure decreased oocyte quality through inducing dysfunctions of mitochondria, ER, lysosomes, Golgi apparatus and ribosome.

During reproduction, oocyte maturation quality is important for successful fertilization and embryo development [24]. The cumulus cell expansion and polar body extrusion are regarded as two important indicators of porcine oocyte maturation [25]. Therefore, we first detected the effects of glyphosate on these two processes. In the present study, the concentration range of glyphosate employed was selected, based on the previous reports on the toxicity effects of glyphosate in *Xenopus laevis* melanophores [26], human peripheral blood mononuclear cells [27], and mouse oocytes [28]. The rate of the PBI extrusion was obviously decreased when oocytes were exposed to 200 μmol/L and 400 μmol/L glyphosate, indicating a failure of porcine oocyte maturation. Altogether, glyphosate exposure impaired oocyte maturation in pigs. A similar study showed the toxic effects of 500 μmol/L glyphosate on mouse oocytes maturation by disrupting germinal vesicle breakdown and polar body extrusion [28]. Another study also confirmed that glyphosate exposure could induce deterioration of metaphase II oocytes and damage of embryos in mice [29]. However, our results are in contrast to the only study performed on effects of glyphosate exposure during IVM on nuclear maturation of porcine oocytes. In that study, 360 μg/mL (about 2130 μmol/L) glyphosate didn’t modify the percentage of oocytes to reach MII stage [6]. Thus, it’s worthy further investigating the toxic mechanism of glyphosate in the present study. In addition, we found that cumulus cell expansion was obvious disrupted after glyphosate exposure, which was also confirmed by the decreased expression of cumulus cell expansion-related genes, *CD44, HAS, TNFAIP6, PTGS1* and *PTGS2*. Our results were consistent with the previous reports showing that *CD44, HAS, TNFAIP6, PTGS* are critical for cumulus cell expansion, that promotes successful oocyte maturation [7, 30–32], which also promoted us to further explore the toxic mechanism of glyphosate during porcine oocyte maturation.

Proper distribution and functions of organelles are critical for oocyte maturation [33]. Since mitochondria are the major organelles to provide energy and regulate intracellular signal transduction during oocyte maturation process, firstly, we analyzed the functions of mitochondria. The results of the present study revealed that mitochondrial distribution, mtDNA copy number as well as mitochondria-related genes (*PGC1α* and *ATP5B*) were disturbed in glyphosate-exposed oocytes. Previous study showed that defects of mitochondria localization could induce mitochondria dysfunction [34]. Additionally, a well-balanced mtDNA copy number is pivotal for mitochondrial functions during oocyte maturation [35, 36]. Thus, mitochondria dysfunction might be the potential incentive considering the toxicity of glyphosate. Similar studies showed the toxic effects of glyphosate on mitochondrial function during mouse oocyte maturation [28, 37]. Our results are also consistent with the previous studies showing that other pesticides, such as paraquat [38] and chlorpyrifos [39], disrupted mitochondrial functions during porcine oocyte maturation. As mitochondria are majors sources of intracellular ROS produced via electron transport in mitochondrial respiratory chains [40], a damaged mitochondrial function might lead to the accumulation of ROS, which will exceed the scavenge ability and induce oxidative stress in cells [41]. Thus, ROS level was then detected in this study to confirm mitochondria dysfunction during porcine oocyte maturation after glyphosate exposure. Our results indicated that the levels of ROS significantly increased after glyphosate exposure. Then as expected, the mRNA levels of antioxidant-related genes (*SOD1*, *SOD2*, *CAT* and *GPX*) observably decreased in glyphosate-exposed oocytes, suggesting oxidative stress in mitochondria and cytoplasm of oocytes. The previous studies on sperm reported that the defective spermatogenesis after glyphosate exposure was related to oxidative stress induced by excessive ROS [42, 43]. ROS and oxidative stress have also been found in oocytes of mice exposed to glyphosate [37]. Furthermore, glyphosate-based herbicides (GBHs) exposure also significantly raised ROS levels during meiotic maturation in mice [5]. Altogether, these results indicated that glyphosate exposure caused mitochondria dysfunction, further inducing oxidative stress in porcine oocytes.

Given that ER plays a pivotal role in oocyte meiotic maturation via its functions on balance of cytoplasmic Ca^2+^ and regulation biogenesis of protein synthesis, folding and maturation, we then analyzed the functions of ER after glyphosate exposure. We first examined the effects of glyphosate on ER distribution, which indicated a disruption of ER accumulation at cortex of oocytes in glyphosate groups. The damaged distribution of ER might indicate the interference of their functions; thus, we then detected the levels of cytoplasmic Ca^2+^, for ER is the major reservoir of intracellular Ca^2+^, and the calcium signaling is one of the most critical communication in oocytes during meiosis [44]. Furthermore, an environmental toxin, Zearalenone, impairs ER functions, leading to the failure of oocyte maturation [23]. Our results showed that the signals of intracellular Ca^2+^ were significantly stronger, indicating dysfunction of ER after glyphosate exposure. It’s also reported that severe hypoxia, oxidative injury, or cytotoxin impaired ER homeostasis by activating ER stress-mediated unfolded protein response (UPR) during reproduction [45, 46]. GRP78, ATF4, and CHOP are related to UPR signaling and ER stress [47]. Among these, GRP78, involved in the folding and assembly of proteins and Ca^2+^ homeostasis, is also a sensor of ER stress [48]. In our present study, we found that the mRNA expression of *GRP78*, *ATF4*, and *CHOP* decreased, and meanwhile, the expression of GRP78 showed a similar downregulation after glyphosate exposure. In addition, we have recently showed that Zearalenone exposure impaired GRP78 expression, resulting in failure of oocyte maturation. Moreover, a previous study has showed that glyphosate induces ER stress in TM3 cells [49]. Therefore, we suggested that glyphosate disrupted the homeostasis and functions of ER, which may further damage porcine oocyte maturation.

Lysosomes, sequester macromolecules submitted by the endocytosis and autophagy pathways for degradation and recycling, plays important roles in regulating oocyte maturation and development [16]. Thus, we also detected the effects of glyphosate on lysosomes. Assessing the distribution of lysosomes, we showed that glyphosate exposure interfered the accumulation of lysosomes at cortex in porcine oocytes, which promoted us to investigate functions of lysosomes. LAMP2, a major protein component of lysosome membrane [50], is critical for lysosome-mediate autophagy in oocytes [51]. It has been reported that ATG7 is prerequisite for induction of autophagy [52], and LC3 is a maker of auto-phagosomes [53]. Our results showed that after glyphosate exposure, the protein expression of LAMP2 was decreased, in accordance with which, the mRNA expression levels of autophagy-related genes, including *LAMP2*, *LC3* and *ATG7* were all reduced. A similar result showed that the expression levels of autophagy-related genes (*LC3, ATG14, mTOR*) and proteins (LC3, ATG12) were significantly decreased in the glyphosate exposed mouse oocytes [28]. Taken together, our data demonstrated that glyphosate exposure induced lysosomal malfunction, which further impaired the maturation quality of porcine oocytes.

Additionally, Golgi apparatus, involved in process, sort and package and transport proteins, is one of the most vital organelles for the development of oocytes [54, 55]. The classical marker of the Golgi apparatus, GM130, plays a key role during mouse oocyte in vitro maturation. Thus, we detected functions of Golgi apparatus after glyphosate exposure as well, and the results showed that glyphosate exposure induced the abnormal distribution of Golgi, however, without affecting the expression of GM130, suggesting that glyphosate exposure might impair Golgi apparatus distribution instead of its functions. Similar to these studies, the previous research showed that exposure to Citrinin (CTN) caused the aberrant Golgi distribution in mouse oocytes [56], and moreover, cigarette smoking condensate (CSC) leads to the fragmentation of Golgi apparatus in lung epithelial cells [57]. Since connections between Golgi and ER are capable of adjusting metabolism to the cellular needs [58], disruption of Golgi apparatus distribution after glyphosate exposure might affect porcine oocyte maturation via damaging homeostasis of Golgi-ER.

Ribosomes, involved in protein synthesis, are critical for oocyte maturation and early embryo development until the initiation of zygotic transcription [59]. To further explore toxic effects of glyphosate on oocytes, we also investigated whether ribosomes functions were disturbed. Ribosome protein S3 (RPS3) was employed as an index of ribosome functions, since RPS3 is a component of the ribosomes and participates in the ribosome maturation [60]. The results showed the levels of RPS3 significantly decreased in glyphosate-exposed oocytes. Similarly, via RNA-seq screening, we previously found malfunction of organelle, including down-regulation of ribosomes biogenesis, was responsible for Ochratoxin A-induced poor quality of oocyte [61]. Thus, our data demonstrated that glyphosate exposure damaged ribosomes functions, further leading to oocyte maturation defects.

## Conclusion

In conclusion, our present study expounds the toxic effects of glyphosate on porcine oocytes. Our results show that glyphosate exposure disrupts functions of mitochondria, endoplasmic reticulum, lysosome, Golgi apparatus and ribosomes, leading to deterioration of oocyte, which may cause reproductive toxicity in their embryo and offspring.

## Data Availability

All data generated or analyzed during this study are included in this published article.

## Abbreviations

COCs: Cumulus-oocyte complexes
ROS: Reactive oxygen species
IVM: In vitro maturation
GV: Germinal vesicle
GVBD: Germinal vesicle breakdown
MI: Metaphase I
MII: Metaphase II
PBI: First polar body
ER: Endoplasmic reticulum
MRL: Maximum residue limits
EGF: Epidermal growth factor
PFF: Porcine follicular fluid
PMSG: Pregnant mare serum gonadotropin
hCG: Human chorionic gonadotropin
PBS: Phosphate-buffer saline
HAS2: Hyaluronan synthase 2
TNFAIP6: Tumor necrosis factor alpha-induced protein
PTGS: Prostaglandin-endoperoxide synthase
CD44: Cluster of differentiation 44
GM130: Golgi matrix protein 130
PGC1α: Peroxisome proliferator activated receptor coactivator 1 alpha
ATP5B: ATP synthase, H^+^ transporting, mitochondrial F1 complex, beta polypeptide
SOD1: Superoxide dismutase 1
SOD2: Superoxide dismutase 2
CAT: Catalase
GPX: Glutathione peroxidase
GRP78: Glucose-regulated protein 78
ATF4: Activating transcription factor 4
CHOP: C/EBP homologous protein
LAMP2: Lysosome-associated membrane protein 2
LC3: Microtubule-associated protein 1 light chain 3
ATG7: Autophagy-related gene 7
RPS3: Ribosomal protein S3
